# Increased CDC20 expression is associated with pancreatic ductal adenocarcinoma differentiation and progression

**DOI:** 10.1186/1756-8722-5-15

**Published:** 2012-04-04

**Authors:** David Z Chang, Ying Ma, Baoan Ji, Yan Liu, Patrick Hwu, James L Abbruzzese, Craig Logsdon, Huamin Wang

**Affiliations:** 1 Departments of Gastrointestinal Medical Oncology, The University of Texas MD Anderson Cancer Center, Houston, TX, USA; 2 Immunology, The University of Texas MD Anderson Cancer Center, Houston, TX, USA; 3 Cancer Biology, The University of Texas MD Anderson Cancer Center, Houston, TX, USA; 4 Melanoma, The University of Texas MD Anderson Cancer Center, Houston, TX, USA; 5 Pathology, The University of Texas MD Anderson Cancer Center, Houston, TX, USA; 6 Virginia Oncology Associates, 1051 Loftis Blvd, Suite 100, Newport News, VA 23606, USA

**Keywords:** CDC20, Pancreatic cancer, Tumorigenesis, Progression, Prognosis

## Abstract

**Purpose:**

Cell division cycle 20 (CDC20) homolog is an anaphase-promoting complex activator that is essential for cell division, but whether its expression in pancreatic ductal adenocarcinoma (PDAC) is significant is unknown. In this retrospective study, we determined whether aberrant CDC20 expression can be used as a biomarker in pancreatic ductal adenocarcinoma (PDAC) tumorigenesis and whether its expression reflects clinical progression.

**Experimental design:**

We compared CDC20 expression levels in normal, cancerous, and inflamed pancreatic tissues from stage II PDAC patients with clinical outcomes and determined CDC20 levels in seven PDAC cell lines. CDC20 was identified using a cDNA microarray database containing gene expression profiles for PDAC tissues and cell lines and chronic pancreatitis and normal pancreas tissues. Its expression was confirmed by real-time quantitative reverse-transcriptase-polymerase chain reaction (qRT-PCR). An immunohistochemical analysis of tissue microarrays from resected PDAC tumors and paired benign pancreatic tissues was done and CDC20 levels were correlated with clinical outcome.

**Results:**

Fifty-six patients were included in this study. A microarray analysis revealed 5-fold higher CDC20 expression in PDAC tissue than in chronic pancreatitis tissue. A qRT-PCR analysis confirmed a mean 20-fold higher CDC20 level in PDAC tissue than in normal pancreas and pancreatitis tissue. RNA and protein CDC20 expression was detected in several PDAC cell lines. An immunohistochemical analysis revealed higher CDC20 protein expression levels in PDAC tissue than in normal pancreas tissue, and high CDC20 expression was associated with poor differentiation (*P *= 0.020) and a significantly lower 5-year recurrence-free survival rate (*P *= 0.039); we also found a trend toward a shorter overall survival duration.

**Conclusions:**

Aberrant CDC20 expression may play an important role in PDAC tumorigenesis and progression and may thus be useful as a marker of disease progression and prognosis and as a therapeutic target.

## Introduction

Pancreatic ductal adenocarcinoma (PDAC) is the fourth leading cause of cancer death in the United States [1]: the annual number of associated deaths is similar to the disease's annual incidence [2]. Similar statistics are reported from other countries. For example, a recent evaluation of the Finnish Cancer Registry, which recorded 4922 patients with pancreatic cancer between 1990 and 1996, revealed that only 89 (1.8%) had survived as long as 5 years [3]. Some exciting progresses have been made for pancreatic cancer; however, there haven't been any breakthrough treatments [4-7]. Pancreatic cancer's poor prognosis is associated with increased cell proliferation and abnormal cell-cycle regulation, although the mechanism and characteristics of this cell growth have not been determined. Thus, prognostic biomarkers and an understanding of their mechanisms are urgently needed to enable further advances in therapeutic modalities and agents. One such marker candidate, cell division cycle 20 homolog (CDC20), is a regulatory protein that is a target molecule in the cell-cycle checkpoint [8].

CDC20 [8] is a component of the mammalian cell-cycle mechanism that activates the anaphase-promoting complex (APC). Its expression is essential for cell division, and its protein activity may be controlled by a balance between ubiquitination and de-ubiquitination. APC activation is required for anaphase initiation and mitosis exit. It is activated during mitosis and G1 phase by CDC20 and HCDH1 [9-14]; these proteins directly bind to APC and activate its cyclin ubiquitination activity. In HeLa cells, CDC20 protein levels and CDC20-APC binding peak in mitosis and decrease drastically in early G1 phase.

Researchers assumed that the CDC20-Mad2-BubR1 complex is short lived and that inactivation of the spindle-assembly checkpoint (SAC) passively releases CDC20 from its inhibitors. However, structural studies of Mad2-CDC20 interaction indicate that Mad2 binds tightly to CDC20 [10-14]. Thus, the release of CDC20 from Mad2 may be an active process. When UbcH10 was added to extracts from cells with an active spindle-assembly checkpoint, the APC/C became active, coincident with CDC20 ubiquitination and Mad2 and BubR1 dissociation [10]. The APC/C and CDC20 complex (APC/CCDC20) is the most downstream target of the SAC [5,7]. Moreover, in the SAC, CDC20 is a critical molecule: it activates the APC and helps dividing cells proceed toward anaphase. CDC20 is highly expressed in many tumor cells, where it results in chromosomal instability. Limited studies have been performed of the mechanism of SAC's response to genotoxic stress. Ectopically expressed p53 or DNA damage-induced endogenous p53 may transcriptionally downregulate CDC20 [12].

High CDC20 expression has been reported in various malignancies [13-16] and many tumor cells [1,14,17,18], including pancreatic cancer cells [15]. However, its clinical significance in human pancreatic cancer is still unknown. Using a cDNA microarray analysis, we noted a 5-fold higher CDC20 expression in PDAC tissue when compared to expression levels in normal pancreas or chronic pancreatitis tissue (unpublished results). These findings suggest that CDC20 may play an important role in tumor development.

The purpose of this study was to determine the significance of aberrant CDC20 expression in PDAC tumorigenesis and progression and whether it will be useful as a biomarker for survival and as a therapeutic target. We compared CDC20 RNA and protein expression in normal, cancerous, and inflamed pancreatic tissue from PDAC patients and compared them with clinical outcomes. We also evaluated CDC20 expression in PDAC cell lines. Our findings suggest that high CDC20 expression is a biomarker for differentiation and recurrence-free survival, which indicate that aberrant CDC20 expression may play an important role in PDAC tumorigenesis and progression and may thus be useful as a marker of disease progression and prognosis and as a therapeutic target.

## Materials and methods

### Patients and tissue samples

For real-time quantitative reverse transcription-polymerase chain reaction (qRT-PCR), we isolated RNA from fresh-frozen samples (five normal pancreatic tissue samples, five pancreatitis tissue samples, six PDAC tissue samples) that had been collected during surgery and stored in the Department of Pathology at The University of Texas MD Anderson Cancer Center (Houston, Texas).

#### Case selection for tissue microarray

We searched the patient record database at MD Anderson to identify patients with stage II PDAC who had undergone pancreaticoduodenectomies between 1990 and 2005 and had not undergone any form of preoperative chemotherapy or radiation therapy. Patients who had undergone preoperative chemotherapy or radiation therapy and those who had died from postoperative complications were excluded from our study. This study was approved by the MD Anderson institutional review board.

Fifty-six patients were included in this study: 39 men and 17 women (median age, 63.7 years [range, 39.8-79.9 years]). Patients' follow-up information through September 2006 was extracted from the medical records and if necessary, updated from a review of the U.S. Social Security Index. Disease recurrence information was updated at each follow-up visit. The recurrence-free survival duration was calculated as the time between the surgery date and first recurrence (if disease recurred) or last follow-up (if disease did not recur) date. Overall survival duration was calculated as the diagnostic biopsy or surgery (if biopsy was not diagnostic) date to death or last follow-up (if death had not occurred) date.

#### Tissue microarrays

To construct the tissue microarrays used in this study, we fixed human tissue samples in formalin and embedded them in paraffin. Archival tissue blocks and their matching hematoxylin-and eosin-stained slides were retrieved, reviewed, and screened by a gastrointestinal pathologist (H.W.) to identify representative tumor regions and non-neoplastic pancreatic parenchyma. For each patient, two cores of tumor and two cores of paired benign pancreatic tissue were sampled from representative areas using a 1.0-mm punch. The tissue microarray was constructed with a tissue microarrayer (Beecher Instruments, Sun Prairie, WI), as described previously [19].

### Cell culture

We determined CDC20 RNA expression in seven pancreatic cancer cell lines (Panc-48, PANC-1, AsPC-1, CFPAC-1, Panc-3, Panc-28, and Capan-2). All cell lines were maintained in Iscove's modified Dulbecco's medium (Life Technologies, Grand Island, NY) containing 10% fetal bovine serum, 2 mM L-glutamine, 50 U/mL penicillin, and 50 mg/mL streptomycin and were maintained in a humidified incubator with 5% CO_2 _at 37°C.

#### RNA extraction, cDNA preparation, and RT-PCR analysis

Total RNA was isolated from cell lines and tissue samples with TRIzol reagent (Invitrogen Life Technologies, Carlsbad, CA), according to the manufacturer's instructions. To measure CDC20 expression, quantitative real-time RT-PCR was performed (sense 5'-GGCACCAGTGATCGACACATTCGCAT-3'; antisense 5'-GCCATAGCCTCAGGGTCTCATCTGCT-3') [9]. PCR was performed under the following conditions: an initial cycle of denaturation at 94°C for 2 minutes, followed by 21-23 cycles of denaturation at 92°C for 45 seconds; annealing at 60°C for 60 seconds; extension at 72°C for 60 seconds; and a final extension at 72°C for 5 minutes. PCR reactions were carried out using a Bio-Rad iCycler iQ5 real-time PCR system (Certified GeneTool, Inc., Milpitas, CA). The 285-bp PCR products were confirmed by agarose gel electrophoresis. As an internal control, we tested the housekeeping gene, ribosomal protein S6 (RPS6), with the primers of RPS6-F (sense 5'-AAGGAGAGAAGGATATTCCTGGAC-3') and RPS6-R (antisense 5'-AAGGGCTTTCTTACAACATACTGG-3'). The primer pairs were designed to generate a DNA fragment spanning an exon-exon junction for RT-PCR analysis.

#### Immunohistochemical CDC20 analysis

Immunohistochemical staining for CDC20 was performed on 4-μm unstained sections from tissue microarray blocks using a mouse monoclonal antibody against CDC20 (Santa Cruz, CA). To determine antigenicity, we treated tissue sections at 100°C in a steamer containing 10 mM citrate buffer (pH 6.0) for 60 minutes. The sections were then immersed in methanol containing 0.3% hydrogen peroxidase for 20 minutes to block endogenous peroxidase activity and incubated in 2.5% blocking serum to reduce nonspecific binding. Sections were incubated for 60 minutes at 37°C with primary anti-CDC20 at a 1:200 dilution. A standard avidin-biotin immunohistochemical analysis of the sections was performed according to the manufacturer's recommendations (Vector Laboratories, Burlingame, CA). Vector red was used as a chromogen, and hematoxylin was used for counterstaining.

#### Image analysis of immunohistochemical signaling

The staining results were evaluated independently by two gastrointestinal pathologists (H.W. and Y.M.). We quantified the percentage of positive cells in each core using the Ariol automated image analysis system (Genetix Corp., San Jose, CA). High CDC20 expression was defined as nuclear staining of ≥ 4.93% of tumor cells, whereas low expression was defined as nuclear staining of < 4.93% of tumor cells or no nuclear staining on the basis of the 75th percentile.

### Statistical analysis

Fisher's exact tests were used to compare categorical data. One-way analysis of variance was used to compare the quantitative real-time PCR data. Overall and recurrence-free survival probability curves were constructed using the Kaplan-Meier method, 95% confidence interval of patient survival and the log-rank test was used to evaluate the statistical significance of differences.

We performed a univariate Cox regression analysis to determine the prognostic significance of patients' clinical and pathologic characteristics. Cox proportional hazards models were fitted for the multivariate analysis. After the interactions between variables had been evaluated, a backward stepwise procedure was used to derive the best-fitting model. The statistical analysis was performed using Statistical Package for Social Sciences software (Macintosh 17.0; SPSS, Inc., Chicago, IL). We used a two-sided significance level of 0.05 for all statistical analyses.

## Results

### CDC20 mRNA expression was significantly higher in PDAC tissue and PDAC cells lines than in normal pancreas and pancreatitis tissue

We used qRT-PCR to determine CDC20 transcript levels in five normal pancreatic tissue samples, five pancreatitis tissue samples, six PDAC tissue samples, and seven pancreatic cancer cell lines (Panc-48, Panc-1, AsPC-1, CFPac-1, Panc-3, Panc-28, and Capan-2). CDC20 mRNA expression was significantly higher in pancreatic cancer tissues (by 21.98-fold) and all exponentially growing cell lines (by 57.68-fold) than in normal pancreas and pancreatitis tissue (Table 1). Post hoc tests revealed no significant differences in CDC20 mRNA levels between normal pancreas and pancreatitis tissues (*P *= 0.420) or PDAC tissues and cell lines (*P *= 0.120). However, we found significant differences between normal pancreas and PDAC tissues (*P *< 0.001), normal pancreas tissue and cell lines (*P *< 0.001), pancreatitis and PDAC tissues (*P *< 0.001), and pancreatitis tissues and cell lines (*P *< 0.001).

**Table 1:** Quantitative RNA CDC20 expression in human PDAC tissue and cell lines

Sample	N	Relative value	ΔCt^a^	P^b^
Normal pancreas	5	1.00	15.70 ± 1.04	-
Pancreatitis	5	1.74	14.90 ± 1.50	0.42
PDAC tissue	6	21.98	11.24 ± 1.87	0.001
PDAC cell lines^c^	7	57.68	9.85 ± 1.52	0.001

a. The difference of cycle number at the threshold level of CDC20 expression; Mean ± Standard deviation

b. one-way ANOVA, pair-wise multiple comparisons test by Student-Newman-Keuls vs. normal pancreas.

c. See Materials and Methods for a list of cell lines used here

### CDC20 protein is highly expressed in PDAC tissue

**Figure 1:**
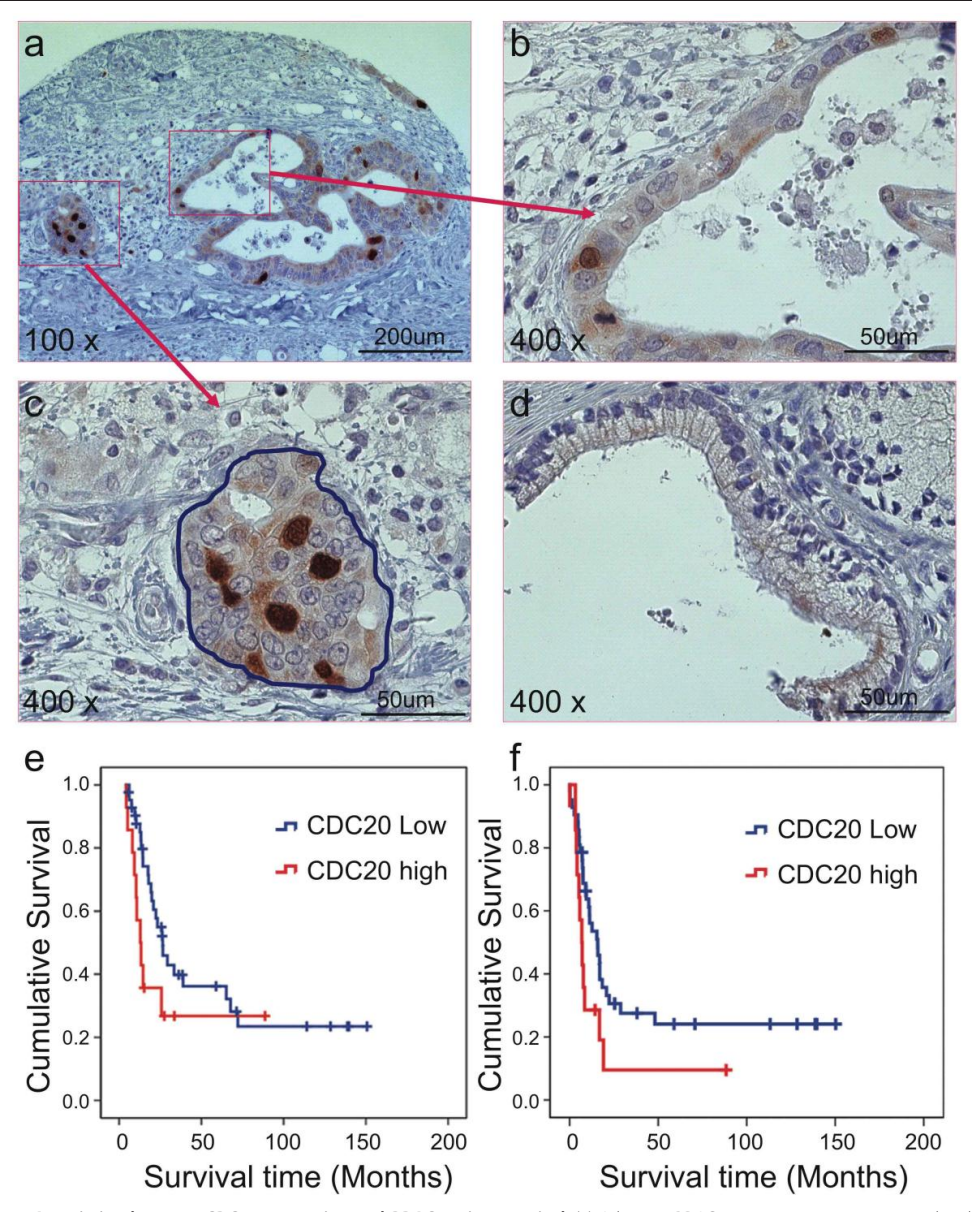
**Association between CDC20 expression and PDAC patient survival**. (**a**) A human PDAC tissue microarray was stained with anti-CDC20 antibody (100×). (**b**) Well-differentiated malignant pancreatic duct with positive CDC20 signaling (400×). (**c**) Poorly differentiated PDAC tissue with positive CDC20 signaling (400×). (**d**) Benign duct with negative CDC20 signaling 400×. (**e**) CDC20 expression was not associated with overall survival in PDAC patients. (**f**) CDC20 expression was associated with recurrence-free survival in PDAC patients. Upper curve, patients with low CDC20 expression; lower curve, patients with high CDC20 expression.

We performed immunohistochemical analyses of tumor tissue and paired benign pancreatic tissue from 56 patients with stage II PDAC. We found extremely low CDC20 protein levels in benign ductal pancreatic tissue (Figure 1d) and much higher levels in PDAC tissue (*P *= 0.048) (Table 2 and Figure 1a-c).

**Table 2:** CDC20 protein expression in PDAC and normal tissue samples

Patient samples	N	CDC20 expression^a^	P^b^
Cancer tissue	56	6.08 ± 11.16	0.048
Benign ductal tissue	53	2.11 ± 2.28	

a. The percentage of positive cells with CDC20 protein expression; Mean ± Standard deviation

b. Student's *t*-test; cancer tissue versus benign tissue.

We compared CDC20 protein expression levels in PDAC tissue samples with patient age; tumor size, differentiation, stage, and margin; lymph node metastasis; and disease recurrence. As shown in Table 3, only tumor differentiation was significantly associated with CDC20 protein expression levels (*P *= 0.020). High CDC20 protein expression in PDAC tissue was associated with poor differentiation (Figure 1c), and low expression was associated with well and moderate differentiation (Figure 1b).

**Table 3:** Association between clinicopathologic characteristics and CDC20 expression

Patient and tumor characteristic	N	CDC20 expression^h^	P
Age (years)			
< 60	17	8.93 ± 17.29	0.449^a^
60-70	24	4.54 ± 6.68	
> 70	15	5.32 ± 7.72	
Lymph node metastasis			
Present	39	6.54 ± 12.86	0.646^b^
Absent	17	5.03 ± 5.78	
Tumor size (cm)			
≤2.0	10	2.98 ± 3.10	0.314^c^
> 2.0	43	7.05 ± 12.50	
Unknown	3	2.50 ± 3.38	
Tumor differentiation			
Well or moderate	41	4.01 ± 5.93	0.020^d^
Poor	15	11.76 ± 18.48	
Tumor stage			
IA and IB	1	19.02 ± N/A	
IIA	16	4.16 ± 4.66	0.482^e^
IIB	37	6.56 ± 13.18	
IV	2	6.15 ± N/A	
Tumor margin			
Negative	45	6.09 ± 11.72	0.993^f^
Positive	11	6.06 ± 8.96	
Tumor recurrence			
None	12	4.76 ± 5.33	0.308^g^
Local and regional	12	2.39 ± 1.97	
Metastasis	32	7.96 ± 14.14	

a. Age range, < 60 versus 60-70 versus > 70 (one-way ANOVA).

b. Lymph node metastasis, present versus absent (Student's *t*-test).

c. Tumor size, ≤ 2.0 cm versus > 2.0 cm (Student's *t*-test)

d. Differentiation grade, well or moderate versus poor (Student's *t*-test).

e. Tumor stage, IIA versus IIB (Student's *t*-test).

f. Tumor margin, negative versus positive (Student's *t*-test).

g. Tumor recurrence, no recurrence versus local and regional recurrence versus metastasis (one-way ANOVA).

h. The percentage of positive cells with CDC20 protein expression; Mean ± Standard deviation

### High CDC20 protein expression is associated with poor recurrence-free survival rates in PDAC patients

The cutoff point was set as 4.93% of positive cells with CDC20 protein expression. High CDC20 expression was associated with a significantly lower 5-year recurrence-free survival rate (*P *= 0.039; Table 4; Figure 1f): 28.57% in patients with low expression versus 14.29% in those with high expression. Patients with low CDC20 expression had a mean survival duration of 45.9 months (95% confidence interval, 26.9-64.9 months), whereas those with high expression had a mean survival duration of 15.6 months (1.8-29.4 months). The trend of the overall survival curve indicates that PDAC patients with high CDC20 expression have a poor overall survival rate; however, because of the small patient sample in our study, this association was not statistically significant (Figure 5e).

**Table 4:** Five-year recurrence-free survival of PDAC patients according to CDC20 expression level

CDC20 expression^a^	N	5-year survival rate (%)	Mean survival duration, months (95% confidence interval)	P^b^
Low	42	28.57	45.9 (26.9-64.9)	0.039
High	14	14.29	15.6 (1.8-29.4)	

a. The percentage of positive cells with CDC20 protein expression; cut off value is 4.93%

b. log-rank (Kaplan-Meier) test, low versus high.

## Discussion

In this study, we found higher CDC20 mRNA expression in human PDAC cell lines and primary PDAC tissues than in normal pancreas tissue from PDAC patients. CDC20 protein over-expressions in human PDAC tissue samples correlated with poor morphological differentiation and short recurrence-free survival of stage II PDAC patients. Based on the role of CDCD20 in regulating mitosis and cell growth [9-11] our findings suggest that CDC20 expression may has a role in maintaining human PDAC cell mitosis and thus facilitating tumor growth.

CDC20 is a regulatory protein, interacting with several other proteins at multiple points in the cell cycle. It is required for two microtubule-dependent processes: nuclear movement prior to anaphase and chromosome separation. As with many other aspects of cell-cycle control, CDC20 degradation is a conserved part of the SAC from yeast to mammals. From its destruction profile, CDC20 can be classified as an early mitotic substrate of the APC/C, as it is degraded during prometaphase, along with cyclin A and Nek2A, even when the SAC is active. Clearly, CDC20's essential function is to activate the APC/C; thus, during unperturbed mitosis, CDC20 protein levels must be maintained [20].

We found high CDC20 RNA and protein expression in PDAC tissue. Thus, a mechanism may exist that involves upregulation of CDC20 RNA production and downregulation of CDC20 protein degradation. Because we did not evaluate CDC20 DNA levels in this study, we could not determine CDC20 region chromosome amplification levels. However, CDC20 expression is suppressed by p53- and p21-dependent genotoxic stresses through cell-cycle-dependent element/cell-cycle gene homology region elements in the CDC20 promoter [16], which may explain CDC20 overexpression. High CDC20 RNA expression in PDAC cells may be induced by mutations of tumor suppressor genes such as p53 and p21. CDC20 is continually synthesized in mitosis and activates the APC/C. However, in the presence of improperly attached kinetochores, Mad2 binds CDC20, which allows it to load onto BubR1. After BubR1 has bound to CDC20, Mad2 is no longer required to sustain the binding and leaves the complex before or after the complex is presented to the APC/C. The APC/C then promotes CDC20 ubiquitylation and degradation to sustain the checkpoint [21]. Given the evidence of an APC/C and CDC20 loop [21], our data suggest that high CDC20 levels in PDAC are due to a dysfunction in APC/C-promoted CDC20 ubiquitylation and degradation.

As shown in Table 3, quantitative CDC20 protein expression levels were significantly associated with PDAC tumor differentiation (*P *= 0.020). Therefore, CDC20 may be a marker for PDAC disease progression.

High CDC20 levels appear to be associated with several other cancers, including serous epithelial ovarian cancer [15], gastric cancer [14], oral cancer [22], and adult T-cell leukemia [23]; high levels are also found in oral squamous cell carcinoma-derived cell lines [22] and the HT29 human adenocarcinoma cell line [13].

CDC20 overexpression has been detected in pancreatic cancer tissue [24] and cell lines [25]. In this study, we also evaluated its association with clinical outcomes. We compared survival rates among PDAC patients whose tumors expressed different CDC20 levels, as assessed by a 4.93% cut-off point (75th percentile of the CDC20 score) for CDC20-positive cells. We found a statistically significant difference in the 5-year recurrence-free survival rate between patients with low and high CDC20 expression (28.57% versus 14.29%; *P *= 0.039), suggesting that CDC20 expression can be used as a prognostic marker and therapeutic target. We also found a trend towards a poorer overall survival rate in patients with high CDC20 expression; this finding should be evaluated further in a larger study population to determine its significance.

We compared CDC20 expression in PDAC tissue with that in normal pancreatic tissue. We found no significant association between high CDC20 expression and lymph node metastasis; tumor size, stage, or margin; or patient age.

There have been development of inhibitors to some of the key intracellular molecules, kinases, e.g., MDM2 (murine double minute 2), ALK (anaplastic lymphoma kinase) and PARP (poly [ADP-ribose] polymerase) as anti-cancer therapeutics [26]. Some of these, for example ALK or PARP inhibitors are already in clinical use. CDC20 could be a potential target for development of anti-cancer agents.

Our results indicate that high CDC20 expression plays an important role in promoting PDAC tumorigenesis. CDC20 may be useful as a marker for disease progression and prognosis and as a therapeutic target that could benefit PDAC patients.

## Competing interests

The authors declare that they have no competing interests.

## Authors' contributions

DZC provided financial support and overall supervision of the project; DZC, CDL, and HW designed the experiments; YM, BJ, and YL performed the experiments; HW provided pathology support and clinical data analysis; JLA, CDL and PH provided scientific advice; YM wrote the initial draft of the manuscript; and all authors provided input on the final version. All authors read and approved the final manuscript.

## Financial support

AACR-PanCAN Career Development Award (H.W.), ASCO-PanCAN Career Development Award (D.Z.C.), and NIH/NCI grant CA128927-01A1 (D.Z.C.).

## References

[B1] Jemal A Siegel R Ward E Murray T Xu J Thun MJ Cancer statistics, 2007 CA Cancer J Clin 2007 57 43 66 10.3322/canjclin.57.1.43 17237035

[B2] American Cancer Society Cancer Facts & Figures 2007

[B3] Carpelan-Holmstrom M Nordling S Pukkala E Sankila R Luttges J Kloppel G Haglund C Does anyone survive pancreatic ductal adenocarcinoma? A nationwide study re-evaluating the data of the Finnish Cancer Registry Gut 2005 54 385 387 10.1136/gut.2004.047191 15710987 PMC1774412

[B4] Javle M Hsueh CT Recent advances in gastrointestinal oncology - updates and insights from the 2009 annual meeting of the American Society of Clinical Oncology J Hematol Oncol 2010 3 11 10.1186/1756-8722-3-11 20331897 PMC2856525

[B5] Hu J Zhao G Wang HX Tang L Xu YC Ma Y Zhang FC A meta-analysis of gemcitabine containing chemotherapy for locally advanced and metastatic pancreatic adenocarcinoma J Hematol Oncol 2011 4 11 10.1186/1756-8722-4-11 21439076 PMC3079694

[B6] Zhang XJ Ye H Zeng CW He B Zhang H Chen YQ Dysregulation of miR-15a and miR-214 in human pancreatic cancer J Hematol Oncol 2010 3 46 10.1186/1756-8722-3-46 21106054 PMC3002909

[B7] Kulke MH Bendell J Kvols L Picus J Pommier R Yao J Evolving Diagnostic and Treatment Strategies for Pancreatic Neuroendocrine Tumors J Hematol Oncol 2011 4 29 10.1186/1756-8722-4-29 21672194 PMC3128039

[B8] Weinstein J Jacobsen FW Hsu-Chen J Wu T Baum LG A novel mammalian protein, p55CDC, present in dividing cells is associated with protein kinase activity and has homology to the Saccharomyces cerevisiae cell division cycle proteins Cdc20 and Cdc4 Mol Cell Biol 1994 14 3350 3363 7513050 10.1128/mcb.14.5.3350-3363.1994 PMC358701

[B9] Fang G Yu H Kirschner MW Direct binding of CDC20 protein family members activates the anaphase-promoting complex in mitosis and G1 Molec Cell 1998 2 163 171 10.1016/S1097-2765(00)80126-4 9734353

[B10] Peters J-M Cell biology: the checkpoint brake relieved Nature 2007 446 868 869 10.1038/446868a 17443175

[B11] Bharadwaj R Yu H The spindle checkpoint, aneuploidy, and cancer Oncogene 2004 23 2016 2027 10.1038/sj.onc.1207374 15021889

[B12] Banerjee T Nath S Roychoudhury S DNA damage induced p53 downregulates Cdc20 by direct binding to its promoter causing chromatin remodeling Nucleic Acids Res 2009 37 2688 2698 10.1093/nar/gkp110 19273532 PMC2677870

[B13] Iacomino G Medici MC Napoli D Russo GL Effects of histone deacetylase inhibitors on p55CDC/Cdc20 expression in HT29 cell line J Cell Biochem 2006 99 1122 1131 10.1002/jcb.21014 16795040

[B14] Kim JMSH Yoon SY Oh JH Yang JO Kim JH Song KS Rho SM Yoo HS Kim YS Kim JG Kim NS Identification of gastric cancer-related genes using a cDNA microarray containing novel expressed sequence tags expressed in gastric cancer cells Clin Cancer Res 2005 11 473 482 15701830

[B15] Ouellet VGM Le Page C Filali-Mouhim A Lussier C Tonin PN Provencher DM Mes-Masson AM Tissue array analysis of expression microarray candidates identifies markers associated with tumor grade and outcome in serous epithelial ovarian cancer Int J Cancer 2006 119 599 607 10.1002/ijc.21902 16572426

[B16] Kidokoro T Tanikawa C Furukawa Y Katagiri T Nakamura Y Matsuda K CDC20, a potential cancer therapeutic target, is negatively regulated by p53 Oncogene 2008 27 1562 1571 10.1038/sj.onc.1210799 17873905

[B17] Takahashi T Haruki N Nomoto S Masuda A Saji S Osada H Identification of frequent impairment of the mitotic checkpoint and molecular analysis of the mitotic checkpoint genes, hsMAD2 and p55CDC, in human lung cancers Oncogene 1999 18 4295 4300 10.1038/sj.onc.1202807 10439037

[B18] Saeki A Tamura S Ito N Kiso S Matsuda Y Yabuuchi I Kawata S Matsuzawa Y Frequent impairment of the spindle assembly checkpoint in hepatocellular carcinoma Cancer 2002 94 2047 2054 10.1002/cncr.10448 11932908

[B19] Eytan E Braunstein I Ganoth D Teichner A Hittle JC Yen TJ Hershko A Two different mitotic checkpoint inhibitors of the anaphase-promoting complex/cyclosome antagonize the action of the activator Cdc20 Proc Natl Acad Sci USA 2008 105 9181 9185 10.1073/pnas.0804069105 18591651 PMC2453698

[B20] Fry AM Yamano H Under arrest in mitosis: Cdc20 dies twice Nat Cell Biol 2008 10 1385 1387 10.1038/ncb1208-1385 19043431

[B21] Nilsson J Yekezare M Minshull J Pines J The APC/C maintains the spindle assembly checkpoint by targeting Cdc20 for destruction Nat Cell Biol 2008 10 1411 1420 10.1038/ncb1799 18997788 PMC2635557

[B22] Thirthagiri E Robinson CM Huntley S Davies M Yap LF Prime SS Paterson IC Spindle assembly checkpoint and centrosome abnormalities in oral cancer Cancer Lett 2007 258 276 285 10.1016/j.canlet.2007.09.008 17959302

[B23] Liu B Hong S Tang Z Yu H Giam CZ HTLV-I Tax directly binds the Cdc20-associated anaphase-promoting complex and activates it ahead of schedule Proc Natl Acad Sci USA 2005 102 63 68 10.1073/pnas.0406424101 15623561 PMC544051

[B24] Li D Zhu J Firozi PF Abbruzzese JL Evans DB Cleary K Friess H Sen S Overexpression of oncogenic STK15/BTAK/Aurora A kinase in human pancreatic cancer Clin Cancer Res 2003 9 991 997 12631597

[B25] Lee YJ Kim EH Jae SL Jeoung D Bae S Seung HK Lee YS HSF1 as a mitotic regulator: Phosphorylation of HSF1 by Plk1 is essential for mitotic progression Cancer Res 2008 68 7550 7560 10.1158/0008-5472.CAN-08-0129 18794143

[B26] Yuan Y Liao YM Hsueh CT Mirshahidi HR Novel targeted therapeutics: inhibitors of MDM2, ALK and PARP J Hematol Oncol 2011 4 16 10.1186/1756-8722-4-16 21504625 PMC3103487

